# Immunotherapy improved cancer related pain management in patients with advanced Hepato-Pancreatic Biliary Cancers: A propensity score-matched (PSM) analysis

**DOI:** 10.3389/fonc.2022.914591

**Published:** 2022-09-21

**Authors:** Xiufang Wu, Fei Qin, Qiangze Zhang, Jianling Qiao, Yulian Qi, Bing Liu

**Affiliations:** ^1^ Department of Pain, Jinan People’s Hospital, Jinan, China; ^2^ Department of Medical Oncology, Jinan Hospital of Integrated Traditional Chinese and Western Medicine, Jinan, China; ^3^ Department of Medical Oncology, Jinan People’s Hospital, Jinan, China; ^4^ Department of Disease Control and Prevention, Rocket Force Characteristic Medical Center, Beijing, China

**Keywords:** PDAC, prognosis, pain, opiod, immunotherapy

## Abstract

**Background:**

Hepato-pancreato-biliary (HPB) cancer is a serious form of cancer. in many HPB cancers, including cholangiocarcinoma (also known as bile duct cancer), pancreatic cancer, hepatocellular carcinoma, gallbladder cancer and ampullary cancer, although several treatment options are developed during these decades, the prognosis is still poor.

**Methods:**

A total of 356 HPB cancers patients in advanced stage received different kinds of treatments including adjuvant chemotherapy, radiotherapy, targeted therapy and immunotherapy. Among these patients with advanced HPB cancers, 135 patients have received standard opioid treatment for pain controlling.

**Results:**

We performed a PSM analysis to minimize differences between groups. Before PSM, 135 patients received standard opioid treatment for pain controlling were enrolled in this study and divided into 4 groups, including chemotherapy, radiotherapy, targeted therapy and immunotherapy. Relevant clinical variables that were available at the time of initial diagnosis were used for 1:1 matching between the two groups. After PSM, the cohort consisted of 18 patients in each group. Prior to PSM, patients received targeted therapy and immunotherapy exhibited shorter median OSs than their counterparts for patients received chemotherapy and radiotherapy (p<0.001). there were so survival differences among all the four different treatments for these patients with HPB cancers (p>0.05). We found the OMED (mg) q/day and NRS scores decreased significantly when patients received immunotherapy treatment. Fewer adverse events were showed between immunotherapy group and other three treatment groups, which was consistent with our previous reports.

**Conclusion:**

In conclusion, we found that given the same survival benefit, immunotherapy reduced opioid consumption in HPB cancers patients and improved the pain management. Moreover, immunotherapy results in fewer other adverse effects.

## Introduction

Hepato-pancreato-biliary (HPB) cancer is a serious form of cancer. Among many HPB cancers, including cholangiocarcinoma (also known as bile duct cancer), pancreatic cancer, hepatocellular carcinoma, gallbladder cancer and ampullary cancer, although several treatment options are developed for these cancers during these decades, the prognosis is still poor. Pancreatic ductal adenocarcinoma (PDAC) ranks the fourth leading cause of cancer-related death with increasing incidence globally (1–3), it is also considered as the most lethal malignancy with the 5-year survival rate lower than 10%. Even for the resectable PDAC patients, the 5-year survival rate is only about 20% (4–7). Hepatocellular carcinoma (HCC) is the sixth most common cancer worldwide, killing more than 782,000 people each year and placing a significant burden on global healthcare systems due to its high prevalence and mortality. A 2012 survey in developing countries showed that hepatocellular carcinoma accounted for 8.1% of all new cancer cases and 83% of all liver cancer cases worldwide. Hepatocellular carcinoma is also the second most common cause of cancer- related death. The American Cancer Society estimates that in 2021, there will be approximately 42,230 new cases of liver cancer in the United States (8–11). Cholangiocarcinoma (CCA) is a malignant tumor originating from bile duct epithelial cells that accounts for approximately 3% of tumors of the digestive system. The treatment effect and clinical prognosis of CCA are not satisfactory. Patients with surgically resected tumors have a 5-year survival rate of 5%, and the survival time of advanced patients who do not receive surgery is less than 1 year. At present, the incidence of CCA is increasing each year (12–15). Unfortunately, most of the patients with HPB cancers are diagnosed with advanced or metastatic disease and supportive cares are vital to the short life span for these patients.

Pain is a common symptom and one of the major burdens on cancer patients (16–18). The prevalence of pain is reportedly 40% for patients after curative treatment, 55% for patients during antitumor treatment and >60% for patients with advanced metastatic cancer. In patients with HPB cancers (19, 20), most of them with advance stages were reported to have cancer related pain and pain management by medicine. Opioids have been the mainstay of current management for moderate to severe cancer pain. There has been no space to debate the utility of opioids for cancer pain treatment. However, concerns are growing about the long-term use of opioids due to unfavorable side effects including tolerance and dependence, sleep disordered breathing, endocrinopathy, cognitive dysfunction and immunosuppression (21–23). In addition, a not-insubstantial percentage of cancer patients reportedly experience opioid-refractory pain. A search for novel strategies to address pain is crucial to improving quality of life for cancer patients. Clinical and preclinical studies have classified cancer pain based on its etiology, such as inflammatory or neuropathic pain. However, etiology-based cancer treatment remains difficult in clinical settings.

In this study, we aimed to compare the different types of adjuvant treatments related with opioids consumption and pain control in patients with late stages of HPB cancers. The treatments included chemotherapy, radiotherapy, drugs based targeted therapy and immunotherapy. Furthermore, we sought to assess related factors in the pain control that influenced the options of cancer treatments.

## Patients and methods

### Patients and treatment schedule

The study was approved by the Ethics Committee of the Institutional Review Board of the Jinan People’s Hospital. Written informed consent was waived owing to the retrospective and deidentified nature of the study. Between February 2015 and February 2020, patients who were pathologically diagnosed with HPB cancers and treated with additional opioid with or without anti-cancers agents as a second- or later-line therapy in Jinan People’s Hospital were enrolled in this retrospective study. All patients had experienced progressive disease after standard first-line treatment. This study was conducted according to the principles of the Declaration of Helsinki. The clinical parameters were collected, including sex, age, stage, presence of liver/brain metastasis, Eastern Cooperative Oncology Group Performance Status (ECOG PS), smoking/drinking history, previous treatment lines and regimens, history of radiotherapy, radiologic and laboratory data.

Next, Postoperative outcomes and treatment included the occurrence of major morbidity (Clavien-Dindo ≥ III) and 30-day mortality. Pathologic parameters were collected according to the 8^th^ edition of the AJCC TNM staging system and included tumor stage, tumor size, extent of lymph node involvement, and tumor grade. Follow-up data for all patients were obtained from their most recent medical review, including documented clinical examination and assessment of computed tomography (CT) scans. Patients’ overall survival (OS) was calculated from the date of the index operation to the date of death or last contact. An independent biostatistician managed and maintained the collected data.

### Outcome definition

Numerical rating scales (NRS) evaluation was used as subjective methods for pain intensity measurement in this study, which was performed by an 11-point NRS and patients were asked to rate their pain on a 0 to 10 scale where 0 indicates “No pain” and 10 “The worst possible pain”. The maximal NRS scores and the mean opioid consumption per day were the primary outcomes of this study. The maximum pain score was extracted from the notes taken by the nurses during the period of hospitalization. This score was plotted to illustrate the pain dynamics during inpatient admission (morning, afternoon, overnight) for patients with all available time points recorded. Narcotic doses were converted to OMEDq/day by using an opioid conversion scale system (24). We calculated the OMED based on morphine-equivalent fractions as described by Jarlbaek et al (25, 26). OMEDq/day was adjusted for patients’ weight(OMEDq/day*Kg), since a large variation in weight was found among patients. Participants were not necessarily using opioids every day during this period.

### Quality of life (QOL) assessments

European organization for the Research and Treatment of Cancer Quality of Life Questionnaire (EORTC QLQ-C30) is widely used scale for cancer patients (27). It includes five functional measures (physical, role, emotional, social, cognitive), eight symptoms (fatigue, pain, nausea/vomiting, appetite loss, constipation, diarrhea, insomnia, dyspnea), as well as global health/QOL and financial impact. Most items use a 4-item scale from ‘not at all’ to ‘very much’ and a one-week recall period. Raw scores are transformed to a 0-100 scale, with higher scores representing better functioning/QOL and greater symptom burden.

### Statistical analyses

Continuous variables were expressed as mean ± SD (standard deviation) and compared using a two-tailed unpaired Student’s t-test. Categorical variables were compared using χ^2^ or Fisher analysis. Life-table estimates of survival time were calculated according to the Kaplan-Meier methodology (28). Potential prognostic variables were analyzed using univariate analyses followed by a multivariate model combining all factors. Results were reported as hazard ratios (HR) with their 95% confidence intervals (CI). A HR > 1 indicated an elevated risk with respect to the reference category. A confidence interval which did not include the value 1 indicated statistical significance at the 5% level (p = 0.05).

## Results

### Patient characteristics

A total of 356 HPB cancers patients in advanced stage received different kinds of treatments including adjuvant chemotherapy, radiotherapy, targeted therapy and immunotherapy with best supportive care. Among these patients with advanced HPB cancers, 135 patients have received standard opioid treatment for pain controlling. Characteristics of all patients received opioid treatment are divided into 4 groups, which are detailed in **Table 1**. Among these patients, there were 49 patients received chemotherapy, 38 patients received radiotherapy, 22 patients received targeted therapy and 26 patients received immunotherapy. There were significant differences in BMI, hospital length of stay, and the proportion of R0 resection between these two groups. Relevant clinical variables that were available at the time of initial diagnosis were used for 1:1 matching between the two groups. After PSM, the cohort consisted of 18 patients in each group for chemotherapy, radiotherapy, targeted therapy and immunotherapy, respectively. The standardized differences after matching were smaller for all background variables compared with those in the unmatched groups such that there were ultimately no significant differences remaining between the groups (**Table 2**).

**Table 1:** Demographics and clinical characteristics of enrolled patients who received opioid treatment before PSM.

Variable	Chemotherapy (n=49)	Radiotherapy (n=38)	Targeted therapy (n=22)	Immunotherapy (n=26)	*P* value
**Sex**					0.752
Female	21	20	10	11	
Male	28	18	12	15	
**ECOG PS**					**0.011**
2	12	15	10	14	
3	37	23	12	12	
**BMI**	25.3 ± 4.3	26.1 ± 5.2	26.4 ± 3.8	25.5 ± 4.3	**0.036**
**Charlson comorbidity Index**					0.281
0	12	11	7	10	
1	21	15	10	9	
2	9	6	3	5	
3	7	5	2	2	
**Tumor and pathologic characteristics**					
**AJCC TNM stage**					0.175
IiI	10	11	8	10	
IV	39	27	14	16	
**Cancer types**					**0.013**
HCC	20	15	12	11	
PDAC	16	16	5	10	
CC	10	6	3	3	
GBC	3	1	2	2	
**Opioids consumption duration (days)**	56.4 ± 12.5	50.2 ± 10.4	59.1 ± 7.6	47.5 ± 5.9	**0.001**

Bold values means p<0.05.

**Table 2:** Demographics and clinical characteristics of enrolled patients who received opioid treatment after PSM.

Variable	Chemotherapy (n=18)	Radiotherapy (n=18)	Targeted therapy (n=18)	Immunotherapy (n=18)	*P* value
**Sex**					0.982
Female	7	8	8	7	
Male	11	10	10	11	
**ECOG PS**					0.847
2	8	9	7	8	
3	10	9	11	10	
**BMI**	25.5 ± 3.7	25.1 ± 4.8	25.7 ± 4.2	25.4 ± 3.7	0.721
**Charlson comorbidity Index**					0.815
0	9	7	8	9	
1	6	8	7	6	
2	2	2	2	2	
3	1	1	1	1	
**Tumor and pathologic characteristics**					
**AJCC TNM stage**					0.916
Iib	6	5	6	6	
III	12	13	12	12	
**treatments types**					0.638
chemotherapy	10	9	10	11	
radiotherapy	4	5	5	4	
targeted therapy	3	3	2	2	
immunotherapy	1	1	1	1	
**Opioids consumption duration (days)**	48.4 ± 8.7	49.2 ± 10.3	49.5 ± 6.7	47.3 ± 5.1	0.217

### Survival analysis of patients with different treatments

**Figure 1:**
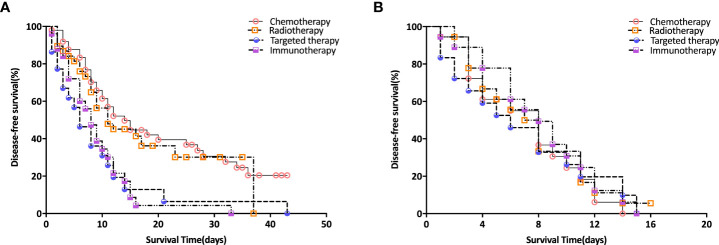
Survival Analysis of Patients with different treatments. **(A)** survival curves before PSM analysis; **(B)** survival curves after PSM analysis.

Prior to PSM, patients received targeted therapy and immunotherapy exhibited shorter median OSs than their counterparts for patients received chemotherapy and radiotherapy (p<0.001) (**Figure 1A**). there were so survival differences among all the four different treatments for these patients with HPB cancers (p>0.05) (**Figure 1B**).

### Comparison of pain management in different groups

**Figure 2:**
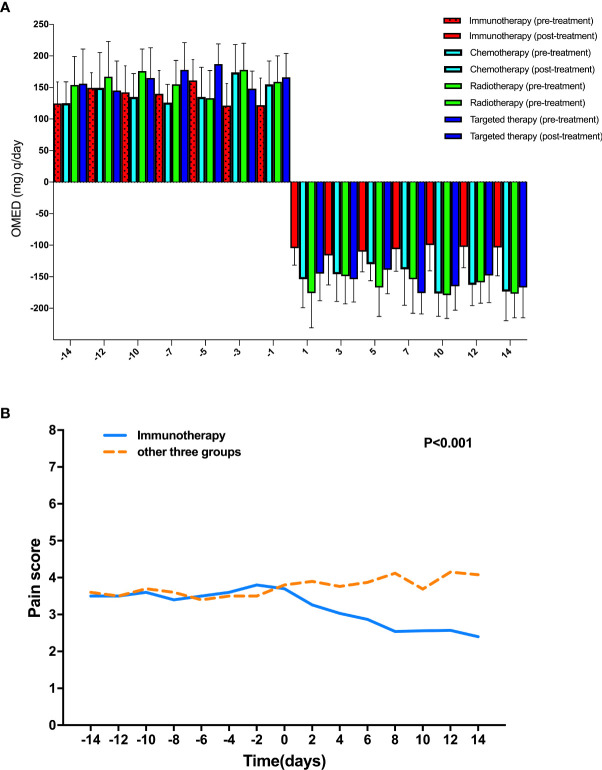
Comparison of pain management in different groups. **(A)** Comparison of OMED (mg) q/day 14 days before and after various treatments after PSM analysis; **(B)** Comparison of NRS scores 14 days before and after various treatments after PSM analysis.

We compared the OMED (mg) q/day and NRS scores 14 days before and after various treatments after PSM analysis. We found the OMED (mg) q/day and NRS scores decreased significantly when patients received immunotherapy treatment (**Figures 1A, B**). Moreover, compared with other three treatment groups, including chemotherapy, radiotherapy and targeted therapy, the OMED (mg) q/day and NRS were significantly lower in the immunotherapy group after treatment (p<0.001) (**Figures 2A, B**).

### Quality of life (QOL) assessments

Fewer adverse events were showed between immunotherapy group and other three treatment groups, which was consistent with our previous reports. In this study. QOL evaluation (EORTC QLQ-C30 v3.0) was performed after PSM analysis. We found several significant factors related to the various treatments for patients with HPB cancers (P <0.05, **Table 3**).

**Table 3:** QOL evaluation (EORTC QLQ-C30 v3.0) in four groups after PSM.

Items	Chemotherapy (n=18)	Radiotherapy (n=18)	Targeted therapy (n=18)	Immunotherapy (n=18)	P value
**Global health status**	56.4	65.4	53.2	46.5	0.004
**Function Scales**					
**Physical functioning**	78.4	66.5	73.2	70.3	0.531
**Role functioning**	65.4	68.3	65.7	60.4	0.527
**Emotional functioning**	72.8	71.2	67.6	70.6	0.805
**Cognitive functioning**	66.5	68.4	65.7	69.6	0.804
**Symptom scales**					
**Fatigue**	51.2	55.4	48.7	43.6	0.549
**Nausea and vomiting**	27.6	26.5	25.3	25.2	0.261
**Pain**	41.2	38.7	43.1	63.7	0.001
**Dyspnoea**	33.2	34.6	35.3	37.3	0.305
**Insomnia**	33.2	34.6	41.2	47.1	0.002
**Appetite loss**	36.5	35.7	35.3	47.1	0.014
**Constipation**	25.7	30.5	39.2	50.9	0.002
**Diarrhea**	7.5	12.6	21.4	28.5	0.015
**Financial difficulties**	45.7	41.5	43.1	52.9	0.471

## Discussion

Cancer pain, in addition to physical pain itself, leads to psychological distress. Cancer pain results from changes in skin, bone, nerve, and other tissues due to direct tumor involvement or metastases, treatment effects (eg, diagnostic procedures, surgery, chemotherapy, radiation therapy), or a combination of these (29–31). The high prevalence of cancer pain and often unfortunate failure to relieve it has resulted in great attention to the barriers that persist. These barriers have been classified as patient, professional, and system obstacles. Targeted attention to each of these barriers can lead to major improvements in the delivery of care. Conventional treatment does not always relieve cancer pain satisfactorily. Although there are conventional treatments for pain relief, many patients with cancer have turned to complementary therapies to help them sustain their physical, emotional, and spiritual well-being.

Cancer immunotherapy, also known as immuno-oncology, is a form of cancer treatment that uses the power of the body’s own immune system to prevent, control, and eliminate cancer. Immunotherapy can: Educate the immune system to recognize and attack specific cancer cells. Boost immune cells to help them eliminate cancer. Anti-programmed cell death protein-1 (PD-1) and programmed cell death protein ligand-1 (PD-L1) agents have remarkably changed the therapeutic strategies in many cancers, such as melanoma, lung cancer, renal cell carcinoma, head and neck cancer and so on. However, anti-PD-1/PD-L1 agents alone only benefit about 20% of unselective cancer patients, and HPB patients marginally benefit from anti-PD-1/PD-L1 agents. Therefore, combination strategy of anti-PD-1/PD-L1 agents plus other therapies has drawn abroad interests. Research have demonstrated that anti-PD-1/PD-L1 agents combined might increase the treatment efficacy of pain control through remodeling the abnormal vasculature and promoting infiltration of immune effector cells into tumors. However, no study has been reported on the comparison of pain management with other treatment in HPB cancers (32–34).

In this study, A proportion of HPB cancers patients remains to be with good performance status enough for second or later line treatment after progression on standard first-line chemotherapy. Currently, chemotherapy was recommended as second or later line treatment commonly for HPB cancers patients, with the median survival. Combination therapeutic strategies that could modify tumor microenvironment, such as molecular targeted therapy, chemotherapy, and radiotherapy, reveal potential effect of overcome resistance to immunotherapy. We performed a PSM analysis to minimize differences between groups. Before PSM, 135 patients received standard opioid treatment for pain controlling were enrolled in this study and divided into 4 groups, including chemotherapy, radiotherapy, targeted therapy and immunotherapy. Relevant clinical variables that were available at the time of initial diagnosis were used for 1:1 matching between the two groups. After PSM, the cohort consisted of 18 patients in each group. Prior to PSM, patients received targeted therapy and immunotherapy exhibited shorter median OSs than their counterparts for patients received chemotherapy and radiotherapy (p<0.001). there were so survival differences among all the four different treatments for these patients with HPB cancers (p>0.05). We found the OMED (mg) q/day and NRS scores decreased significantly when patients received immunotherapy treatment. Fewer adverse events were showed between immunotherapy group and other three treatment groups, which was consistent with our previous reports.

In conclusion, we found that given the same survival benefit, immunotherapy reduced opioid consumption in HPB cancers patients and improved the pain management. Moreover, immunotherapy results in fewer other adverse effects.

## Data availability statement

The original contributions presented in the study are included in the article/supplementary material. Further inquiries can be directed to the corresponding author.

## Ethics statement

The studies involving human participants were reviewed and approved by Jinan People’s Hospital. The ethics committee waived the requirement of written informed consent for participation.

## Author contributions

Conceived and designed the experiments: XW, BL; Performed the experiments: FQ, QZ, JQ, YQ; Statistical analysis: XW, YQ; Wrote the paper: XW, BL. All authors read and approved the final manuscript.

## Conflict of interest

The authors declare that the research was conducted in the absence of any commercial or financial relationships that could be construed as a potential conflict of interest.

## Publisher’s note

All claims expressed in this article are solely those of the authors and do not necessarily represent those of their affiliated organizations, or those of the publisher, the editors and the reviewers. Any product that may be evaluated in this article, or claim that may be made by its manufacturer, is not guaranteed or endorsed by the publisher.
